# *Kingella kingae* Endocarditis and Septic Pulmonary Emboli in a 12‐Year‐Old Male With Repaired Tetralogy of Fallot and a Bioprosthetic Pulmonary Valve

**DOI:** 10.1155/cric/2583276

**Published:** 2026-02-20

**Authors:** Cyndee Jocson, Steffan Sernich, Patrick Nelson, Timothy Pettitt

**Affiliations:** ^1^ Department of Pediatric Cardiology, Manning Family Children′s, Louisiana State University Health Sciences Center, New Orleans, Louisiana, USA, lsu.edu; ^2^ Department of Pediatric Cardiology, Nemours Children′s Health, University of Central Florida, Orlando, Florida, USA, ucf.edu; ^3^ Division of Pediatric Cardiothoracic Surgery, Manning Family Children′s, New Orleans, Louisiana, USA; ^4^ Division of Pediatric Cardiothoracic Surgery, Manning Family Children′s, Louisiana State University Health Sciences Center, New Orleans, Louisiana, USA, lsu.edu

**Keywords:** bioprosthetic valve, endocarditis, *Kingella kingae*, septic emboli

## Abstract

*Kingella kingae*, typically known for causing osteoarticular infections in young children, is an emerging cause of infective endocarditis (IE) in older children with congenital heart disease. This report describes a 12‐year‐old with repaired tetralogy of Fallot and a bioprosthetic pulmonary valve who developed culture‐negative IE due to *K. kingae*, confirmed by microbial cell‐free DNA testing and valve tissue PCR. His course was complicated by septic pulmonary emboli, requiring valve replacement. The case highlights the role of molecular diagnostics in managing culture‐negative IE and the need for timely surgical intervention in the presence of embolic complications.

## 1. Background

*Kingella kingae* is a recognized cause of infective endocarditis (IE) in the pediatric population, particularly among children with underlying congenital heart disease (CHD) or prosthetic valves. *K. kingae* can invade the bloodstream following mucosal disruption and adhere to endocardial surfaces, causing infection. The organism′s fastidious growth often results in negative cultures, contributing to diagnostic delays. Recent reports underscore the importance of molecular assays in improving detection and highlight that *K. kingae* IE, whereas rare, can lead to severe complications including valvular destruction and systemic embolization if not promptly identified and treated [1, 2].

## 2. Case Presentation

**Figure 1:**
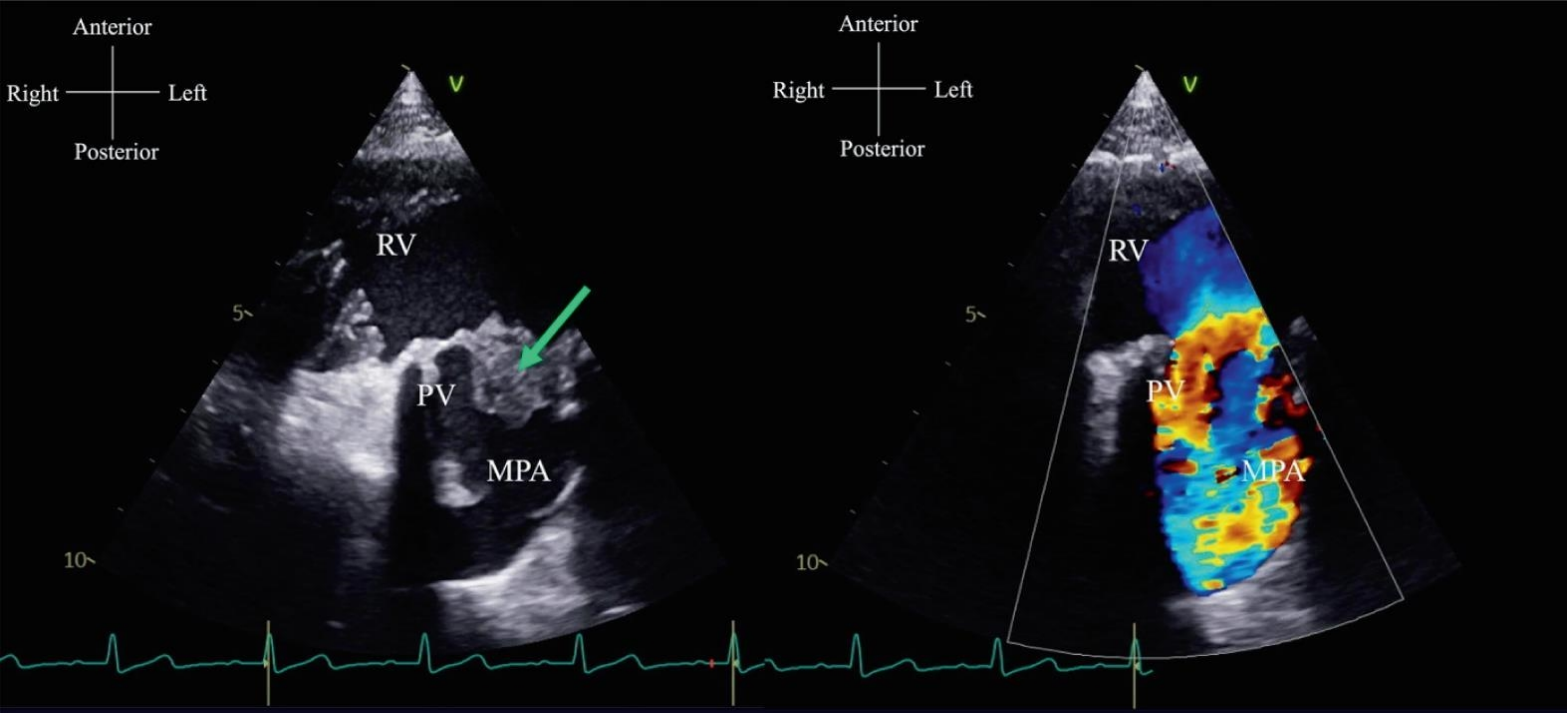
TTE in parasternal short axis view showing a large vegetation (arrow) on the bioprosthetic PV in 2D (left) and mild PV stenosis in color (right). (RV = right ventricle, PV = pulmonary valve, MPA = main pulmonary artery).

A 12‐year‐old male with repaired tetralogy of Fallot and a bioprosthetic pulmonary valve (PV) presented with a 10‐day history of fever, decreased appetite, and fatigue. He had recently recovered from a mild febrile illness associated with oral ulcerations. There was no history of recent dental procedures or other surgeries. In the emergency department, he was afebrile and hemodynamically stable, with vital signs appropriate for age. Cardiac examination revealed a grade 1/6 systolic ejection murmur best heard on the left upper sternal border. He was found to have significantly elevated inflammatory markers including procalcitonin of 7.04 ng/mL, C‐reactive protein of 29.8 mg/dL, erythrocyte sedimentation rate of 85 mm/h, fibrinogen of 556 mg/dL, and D‐dimer of 4.01 FEU. Notably, there was no leucocytosis. Blood cultures were obtained. A transthoracic echocardiogram (TTE) revealed a large vegetation on the PV accompanied by mild pulmonary regurgitation (Figure 1).

**Figure 2:**
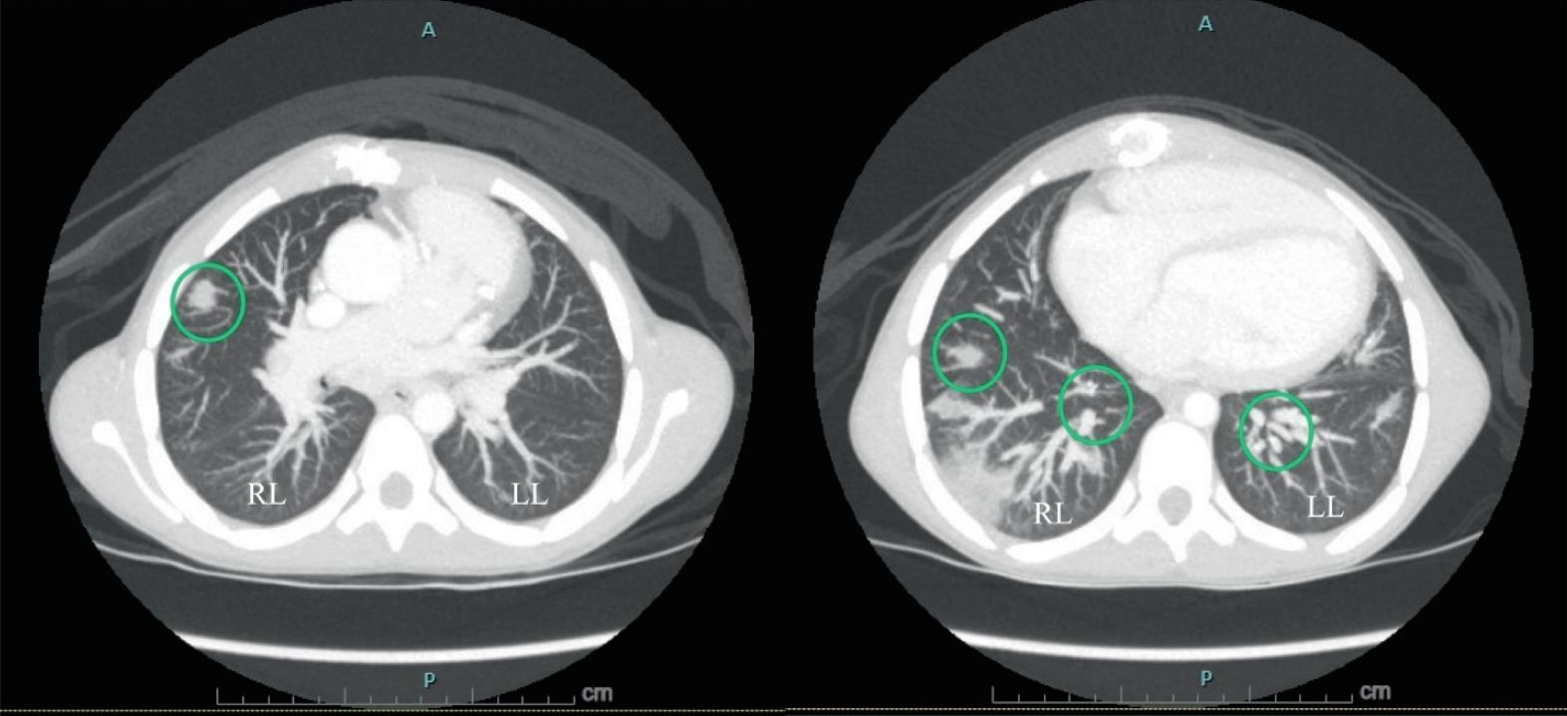
Contrast CT scan of the chest in axial plane showing bilateral multifocal septic pulmonary emboli (circles). (RL = right lung, LL = left lung).

The patient was started on vancomycin (60 mg/kg/day divided every 6 h) and cefepime (150 mg/kg/day divided every 8 h). He was admitted to the pediatric cardiac unit, and the infectious disease service was consulted. Daily blood cultures were obtained, and an expanded infectious work‐up was initiated. All cultures remained negative. However, microbial cell‐free DNA testing returned strongly positive for *K. kingae*, with a quantified load exceeding 316,000 mol/*μ*L. Antibiotic therapy was subsequently narrowed to ceftriaxone (50 mg/kg twice daily). Inflammatory markers showed a downward trend, indicating a partial response to treatment. Despite this, on hospital day 6, he continued to have fevers and began experiencing chest and back pain. Chest computed tomography (CT) scan revealed bilateral multifocal septic emboli (Figure 2).

**Figure 3:**
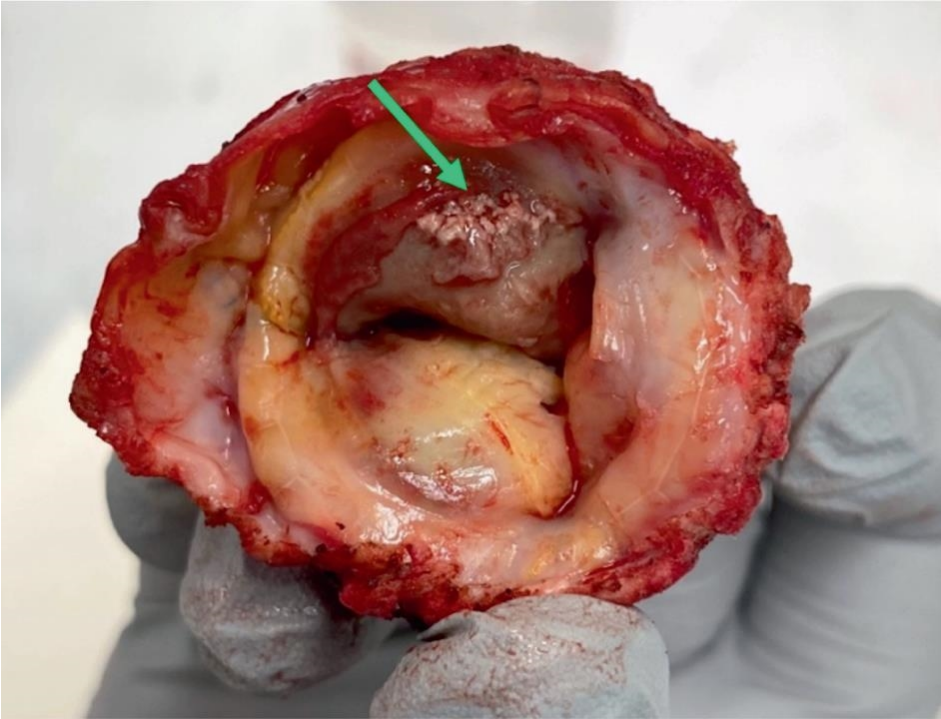
Gross specimen of the bioprosthetic valve showing darkly discolored vegetative irregularities and fibrinopurulence (arrow).

**Figure 4:**
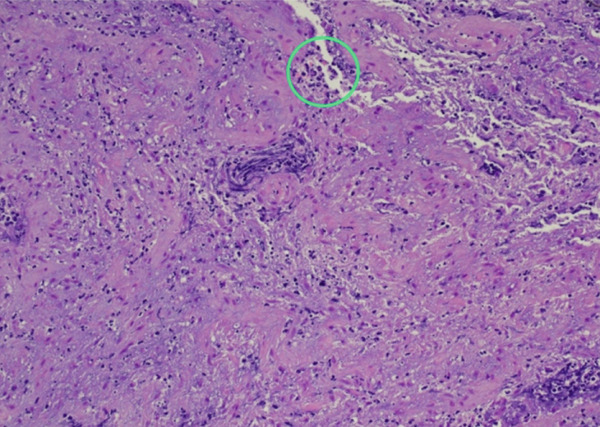
Microscopic specimen showing valvular tissue with focal calcification and neutrophilic inflammation (circle).

A continuous heparin infusion was initiated. The pediatric cardiothoracic surgery team was consulted, and on hospital day 11, the patient underwent PV replacement with a 28 mm homograft. The procedure was completed without complications. Surgical specimens were sent for pathologic evaluation, which showed necrotic and calcified valvular tissue with acute inflammation on histology (Figures 3 and 4). PCR testing of the valve tissue was positive for *K. kingae*.

Postoperatively, the patient was managed in the cardiac intensive care unit. His recovery was complicated by transient hypertension requiring a brief nicardipine infusion. A peripherally inserted central catheter (PICC) line was placed to facilitate completion of a 6‐week course of intravenous antibiotics. The patient was discharged on hospital day 17 with arrangements for weekly laboratory monitoring and PICC line care.

## 3. Discussion

A targeted narrative review was conducted using PubMed (2000–2025), focusing on pediatric *K. kingae* endocarditis and molecular diagnostic techniques. *K. kingae* is a facultative anaerobic, *β*‐hemolytic, gram‐negative coccobacillus and a member of the HACEK (*Haemophilus* species, *Aggregatibacter* species, *Cardiobacterium hominis*, *Eikinella corrodens*, *Kingella* species) group [1, 2]. Invasive *K. kingae* infections primarily affect children between 6 months and 4 years of age, most commonly presenting as osteoarticular infections or bacteremia. Additionally, *K. kingae* is responsible for approximately 3%–5% of all IE cases, with the majority occurring in infants and young children, particularly those with CHD or rheumatic valvular abnormalities [2, 3].

The organism initially adheres to the respiratory epithelium, enabling colonization of the oropharynx. Following colonization and breach of the epithelial barrier, dissemination to target sites, such as bone, joints, or endocardium, occur hematogenously, requiring the bacterium to survive the hostile intravascular environment and successfully establish infection in normally sterile tissues [4, 5]. Unlike other invasive *K. kingae* infections, endocarditis typically manifests with high‐grade fever, leukocytosis, and significantly elevated inflammatory markers. One of the most severe complications of *K. kingae* endocarditis is embolic dissemination, which can result in serious neurological outcomes. Additional reported complications include valvular perforation, severe regurgitation, heart failure, cardiogenic shock, meningitis, brain abscess, and death [1, 2, 5].

*K. kingae* is notoriously difficult to isolate using conventional culture techniques, particularly in pediatric cases where bacteremia is often of low density. Successful isolation typically requires specialized media, extended incubation, and optimal specimen handling, which frequently lead to false‐negative results. In contrast, nucleic acid amplification assays have demonstrated superior diagnostic yield and markedly shorter turnaround times, providing organism identification within hours compared with the 48–72 h required for culture. The rapidity and sensitivity of PCR‐based methods are especially valuable in children, where early recognition directly influences antimicrobial and surgical decision‐making [1, 5, 6]. Once *K. kingae* endocarditis is diagnosed, the recommended antibiotic therapy is a third‐generation cephalosporin, typically ceftriaxone, administered for 4 weeks for native valves and 6 weeks for prosthetic valves [2, 6].

According to the 2016 consensus guidelines from the American Association for thoracic surgery, surgical intervention for IE is indicated in patients presenting with heart failure, severe valvular dysfunction, prosthetic valve endocarditis, invasive infection such as paravalvular abscess or cardiac fistula, recurrent systemic embolization, large mobile vegetations, or persistent sepsis despite appropriate antibiotic therapy [7]. Notably, pediatric surgical guidelines for IE remain largely extrapolated from adult data due to the scarcity of pediatric‐specific studies. Moreover, the optimal timing of surgical intervention for *K. kingae* endocarditis in patients with CHD or prosthetic valves remains undefined, with no established consensus to guide clinical decision‐making [2].

## 4. Conclusion

*K. kingae* is an important but often underrecognized cause of IE, particularly in children with CHD. Its fastidious growth characteristics frequently result in culture‐negative presentations, delaying diagnosis and targeted therapy. In cases involving prosthetic valves, the rapid progression and risk of septic embolization present significant management challenges. Early identification and timely surgical intervention are critical to prevent irreversible valve damage and systemic complications.

## Funding

No funding was received for this manuscript.

## Consent

Written informed consent was obtained from the patient′s legal guardian for publication of this case and accompanying images.

## Conflicts of Interest

The authors declare no conflicts of interest.

## Data Availability

The data that support the findings of this study are available from the corresponding author upon reasonable request.
